# The efficacy of etanercept as anti-breast cancer treatment is attenuated by residing macrophages

**DOI:** 10.1186/s12885-020-07228-y

**Published:** 2020-09-03

**Authors:** Elnaz Shirmohammadi, Seyed-Esmaeil Sadat Ebrahimi, Amir Farshchi, Mona Salimi

**Affiliations:** 1 School of Pharmacy, International Campus, Tehran University of Medical Sciences, Tehran, Iran; 2 Biopharmaceutical Research Center, AryoGen Pharmed Inc., Alborz University of Medical Sciences, Karaj, Iran; 3 Physiology and Pharmacology Department, Pasteur Institute of Iran, P.O. Box: 13164, Tehran, Iran

**Keywords:** TNF-α, Etanercept, Breast cancer, Macrophages, Systems biology

## Abstract

**Background:**

Interaction between microenvironment and breast cancer cells often is not considered at the early stages of drug development leading to failure of many drugs at later clinical stages. Etanercept is a TNF-alpha inhibitor that has been investigated for potential antitumor effect in breast cancer with conflicting results.

**Methods:**

Secretome data on MDA-MB-231 cancer cell-line were from public repositories and subjected to gene enrichment analyses. Since MDA-MB-231 cells secrete high levels of Granulocyte-Monocyte Colony Stimulating Factor, which activates macrophages to promote tumor growth, the effect of macrophage co-culturing on anticancer efficacy of Etanercept in breast cancer was evaluated using the Boolean network modeling and in vitro experiments including invasion, cell cycle, Annexin PI, and tetrazolium based viability assays and NFKB activity.

**Results:**

The secretome profile of MDA-MB-231 cells was similar to the expression of genes following treatment of breast cancer cells with TNF-α. Accordingly, inhibition of TNF-α by Etanercept decreased MDA-MB-231 cell survival, induced apoptosis and cell cycle arrest in vitro and inhibited NFKB activation. The inhibitory effect of Etanercept on cell viability, cell cycle progression, invasion and induction of apoptosis decreased following co-culturing of the cancer cells with macrophages. The Boolean network modeling of the changes in the dynamics of intracellular signaling pathways revealed NFKB activation by secretome of macrophages, leading to a decreased efficacy of Etanercept, suggesting NFKB inhibition as an alternative approach to inhibit cancer cell growth in the presence of macrophage crosstalk.

**Conclusion:**

This study indicates that the effect of Etanercept may be influenced by residing macrophages in tumor microenvironment, and suggests a method to predict the effect of drugs in the presence of stromal cells to guide experimental designs in drug development.

## Background

Crosstalk between cancerous and stromal cells in an inflammatory-based microenvironment not only affects the aggressive behavior of cancer cell, but also influences the efficacy of anti-cancer drugs [1–4]. On the other hand, drug impacts are usually assayed in cell culture or immunosuppressed mice models [5], neither of which provides a clear picture of the complicated interactions between tumor cells and their surrounding inflammatory stroma, eventually resulting in inconsistencies in the outcome of clinical trials [6]. It is thus naïve to assess the sole effect of inhibiting a drugable target in the aggressive cancer cells while ignoring the context of micro-environmental changes [4]. Communication between cancer cells and their surrounding stroma is through secretion by these cells of soluble factors including growth factors, chemokines and cytokines [7]. The secreted factors reprogram the surrounding stroma with the aim of neutralizing the impact of various intruders disrupting the survival of the cancer cells [8, 9].

One of the most influential cytokines in tumor microenvironment is Tumor Necrosis Factor-alpha (TNF-α) [10, 11], long known for its dual effects cancer [12]. Depending on the cellular context as well as its concentration, the effects of TNF-α may vary from inducing necrosis to survival benefits, endowing invasive properties and driving epithelial-mesenchymal transition (EMT) [13].

Considering the pleiotropic and conflicting effects of TNF-α, various efforts have been made to exploit this cytokine as a therapeutic target in cancer. In this regard, some approaches have used TNF-α administration to tumor tissue to induce cell death due to its necrotizing effects [14]. However, using TNF-α as a necrotizing agent has been associated with significant lethal side effects limiting the applicability of TNF-α administration to patients [15]. On the other side, TNF-α activates NFKB transcription factor along with JNK and p38 in a cellular context-dependent manner [16]. This canonical pathway induces the expression of several downstream targets of NFKB leading to the increased invasiveness of cancer cells. A different approach that has attracted a great of attention is the use of TNF-α inhibitors, such as Etanercept, as candidates for cancer biotherapy [17].

Etanercept is a dimeric soluble receptor for TNF-α, which competes with the cell surface receptors For TNF binding. A phase II clinical trial evaluating the therapeutic efficacy of Etanercept in patients with advanced metastatic breast cancer revealed a reduction in the concentration of TNF-α related cytokines such as CCL2; however, disease stabilization was detected in only one patient [18]. In order to elucidate the underlying reason for this outcome, we aimed at integrating molecular-cellular experiments with systems modeling approaches to compare the effect of Etanercept in breast cancer cells alone and in the context of host immune cells.

## Methods

### High through-put data analysis and enrichment-based test*s*

Proteomics data providing the secretome of MDA-MB-231 cells were extracted from GSE51938 dataset in GEO. The secretion profile was submitted to Enrichr online web (http://amp.pharm.mssm.edu/Enrichr/). “LINCS L1000 Ligand Perturbations up” library was used for enrichment analysis. This library consists of gene expression changes induced by various ligands (e.g. growth factors and cytokines) and displays ligands inducing similar gene expression profiles.

Gene-set enrichment tests including gene ontology (GO) biological process, transcription factors and signaling pathways were carried out using the corresponding libraries embedded in Enrichr online web tool.

### Boolean network simulation

Cellular signaling network was constructed based on the literature survey [19] and translated into Boolean rules. Asynchronous simulation mode was used to predict the effect of macrophages on the anti-cancer efficacy of Etanercept. BoolNet R package was used to perform the dynamic modeling and analysis of attractor states [20].

### Cell culture and condition medium preparation

MDA-MB-231 human breast cancer cell line and THP-1 human monocytic cells were purchased from Pasteur Institute of Iran (Tehran, Iran). A total of 2 × 10^5^ THP-1 cells were seeded in 2 ml RPMI supplemented with 10% FBS in the upper chamber of 6-well insert plates (SPL Life Sciences, Seoul, South Korea). Cells were treated with 10 ng/ml of PMA (Phorbol 12-myristate 13-acetate) for 24 h to allow differentiation of THP-1 cells to M0 macrophages and their subsequent attachment to the inserts. After 24 h, 2 × 10^5^ of MDA-MB-231 cells were seeded in the lower chamber in 2 ml of DMEM. The co-culturing system was kept in 5% CO2 incubator for 72 h.

To prepare condition medium (CM), the upper insert was withdrawn and MDA-MB-231 cells were washed 3 times with PBS and starved for 24 h in the serum free DMEM. The condition medium was then filtered using 0.2 μm filter and kept at − 70 °C until use. CM was diluted with 50% of fresh medium for further experiments.

### Tetrazolium-based viability test

Co-culturing of MDA-MB-231 with THP-1 cells was performed as described in the previous section. Co-cultured as well as MDA-MB-231 cells were treated with 1 or 10 μg/ml of Etanercept (Aryogen Pharmed, Tehran, Iran) or control buffer for 72 h. The concentration of Etanercept was selected based on the plasma concentration obtained after weekly administration of the drug to the arthritis rheumatoid patients [7]. After 72 h, THP-1 containing inserts were discarded, and 20 μl of 5 mg/ml of MTT (3-(4,5-dimethylthiazol-2-yl)-2,5-diphenyltetrazolium bromide) stock solution was added to each well. Following 3 h of incubation, the supernatants were aspirated, and 200 μl of dimethylsulfoxide (DMSO) were used to dissolve purple formazan in each well. Absorbance was measured at 545 nm using microplate Reader (Stat Fax-2100, ST. Louis, USA).

### Cell cycle analysis

MDA-MB-231 cells were harvested after 72 h of co-culturing with the differentiated macrophages in the presence and absence of 1 or 10 μg/ml Etanercept. Cells were washed 3 times with cold PBS, detached using 0.025% trypsin and re-suspended in 70% ethanol and stored at − 20 for at least 24 h to allow cell fixing. Cells were pelleted by centrifugation at × 400 *g* and ethanol was discarded. They were then stained with 200 μl of PI (Propidium Iodide) (1 mg/ml) stock solution supplemented with 150 μl of stock solution of DNAse-free RNAse A (CinaClone, Tehran. Iran) (2 mg/ml) and 0.1% Triton X-100 in 10 ml PBS, and then analyzed by Flow cytometry (PARTEC GmbH, Munster, Germany). FlowJo software was used for data analysis and measuring fraction of the cells in four stages of the cell cycle, i.e. G1, S, G2 and subG1.

### Annexin-PI apoptosis assay

To measure the percentage of apoptotic cells induced by Etanercept either in MDA-MB-231 cells alone or co-cultured with the macrophages, Annexin-V staining with PI was used based on the manufacture’s instruction. Briefly, cells were washed with PBS and harvested with 0.025% trypsin. Detached cells were centrifuged at 400×*g* for 5 min and re-suspended in Annexin-PI solution containing 2 μl annexin-V-FLUOS labeling agent, 2 μl PI solution and 1 ml incubation buffer, and incubated at 37 °C for 15 min, and then analyzed by flow cytometry.

### NFKB activity determination

A total of 1 × 10^6^ MDA-MB-231 cells were seeded into 100 mm cell culture plates and allowed to attach for 24 h. Cells were then treated with 50% diluted CM or normal medium as control for 72 h. Activity of NFKB was determined using TransAM® NFκB Transcription Factor ELISA Kit (#40096, Active Motif, Belgium) as described in the kit manual. In brief, following the incubation period, cells were washed twice with PBS supplemented with PhosStop phosphatase inhibitor cocktails (Roche, USA) and 0.05% NP-40. Ultra-centrifugation was used to extract nucleus from the cytoplasmic fraction. The nucleus pellet was further treated with nucleus lysis buffer and centrifuged at × 14,000 *g* to extract nuclear proteins. Protein assay was performed using Bradford method [21], and 10 μg of nuclear extract was added to each well of DNA-coated wells. To calculate the fraction of DNA-attached NFKB, HRP-conjugated anti-phospho-p65 primary antibody was added to each well and the fluorescence intensity was measured at 630 nm (Stat Fax-2100, ST. Louis, USA).

### Quantitative real time PCR for the analysis of macrophage cytokines

A total of 1 × 10^6^ of differentiated THP-1 cells using10 ng/ml of PMA were treated with CM for 48 h and incubated at 37 °C. Cells were washed with PBS and lysed using RNX - Plus solution (CinaClone, Tehran, Iran). Total mRNA was extracted and subjected to cDNA synthesis using Revert Aid first strand cDNA synthesis kit (ThermoFisher Scientific, USA) according to the manufacturer’s instructions. PCR was carried out using Taqman mastermix [21]. Forward (F) and reverse (R) primer sequences used were as following:

TNF-Α-F: 5′-CCCTGACATCTGGAATCTGGAG-3′,

TNF-Α-R: 5′-TCAAGGAAGTCTGGAAACATCTGG-3′,

CCL22-F: 5′-TGGGTGAAGATGATTCTCAATAAGC-3′,

CCL22-R: 5′-CTATAATGGCAGGGAGGTAGGG-3′,

IL10-F: 5′-CTTGCTGGAGGACTTTAAGGGTTAC-3′,

IL10-R: 5′-CTTGATGTCTGGGTCTTGGTTCTC-3′.

ΔΔCT was calculated and normalized according to the expression of GAPDH using the following primers, GAPDH-F: 5′-ACATCAAGAAGGTGGTGAAGCAG-3′, GAPDH-R: 5′- GCGTCAAAGGTGGAGGAGTG-3.

### Matrigel invasion assay

MDA-MB-231 cells were grown in CM for 72 h. Cells were washed 3 times with PBS and starved with serum-free DMEM for 24 h. 24-well insert plates (SPL Life Sciences, Seoul, South Korea) with 8 μm pore size were coated with 1 mg/ml of matrigel (BD Biosciences, San Jose, USA). After 4 h of incubation of the coated plates at 37 °C, when a smooth layer was formed on the insert, a total of 1 × 10^5^ starved cells were added to the upper chamber of each well in serum free medium, and the lower chamber was supplemented with DMEM containing 10% FBS as attractant. After 24 h, the inserts were fixed in 4% formaldehyde and stained using 0.05% crystal violet. Traversed cells across the membrane were visualized under an inverted microscope.

### Statistical analysis

For the enrichment tests, adjusted *p*-value by Bonferroni method < 0.05 was considered as significant. For experimental assays, GraphPad Prism was used for data analysis. Data represent mean ± S.E.M from the indicated replicates for each test. ANOVA was used to determine the significance of difference (*p*-value < 0.05).

## Results

### In silico analysis of breast cancer cells secretome

Considering the importance of cytokine profile of invasive breast cancer cells in communicating with stromal cells, the secretome of mesenchymal-like breast cancer cells (MDA-MB-231 cells) were initially extracted from the work of Su et al. [22]. The authors used RayBio® Cytokine Antibody Array and provided their data freely available in GEO database (GSE51938).

The secreted cytokines with an intensity greater than 0.2 were chosen for enrichment-based tests using the Enrichr online web tool [23]. Using up-regulated signatures induced by ligand perturbations in LINCS L1000 module within Enrichr, the list of secreted cytokines from MDA-MB-231 was found to be statistically mimicked when breast cancer cells were treated with IL1 and TNF-α (Table 1). Moreover, the transcription factor enrichment analysis on ChEA database using Enrichr web tool revealed RELA (a component of NFKB transcription factor) as the top enriched transcription factor associated with the secretome of MDA-MB-231 cells (FDR adjusted *p*-value < 0.05). These results suggested NFKB as being highly active in the invasive breast cancer cells resulting in the secretion of a number of cytokines similar to those secreted by cells treated with TNF-α. They also provided a rationale for using clinically available TNF-α inhibitors as an approach to alter the secretion profile of invasive breast cancer cells.

**Table 1:** Enrichment of MDA-MB-231 cell line secretome against “up-regulated gene perturbations induced by ligands in LINCs1000” and CHEA library using Enricher web tool

**Index**	**Name**	**Adjusted** ***p*-value**
1	IL1-BT20	0.0002643
3	TNFA-BT20	0.0002643
5	IL1-MDAMB231	0.0002643
7	TNFA-MDAMB231	0.003165
1	RELA_24523406_ChIP-Seq_FIBROSARCOMA_Human	0.004114

### Anti-TNF therapy inhibited growth of breast cancer cells

**Fig. 1:**
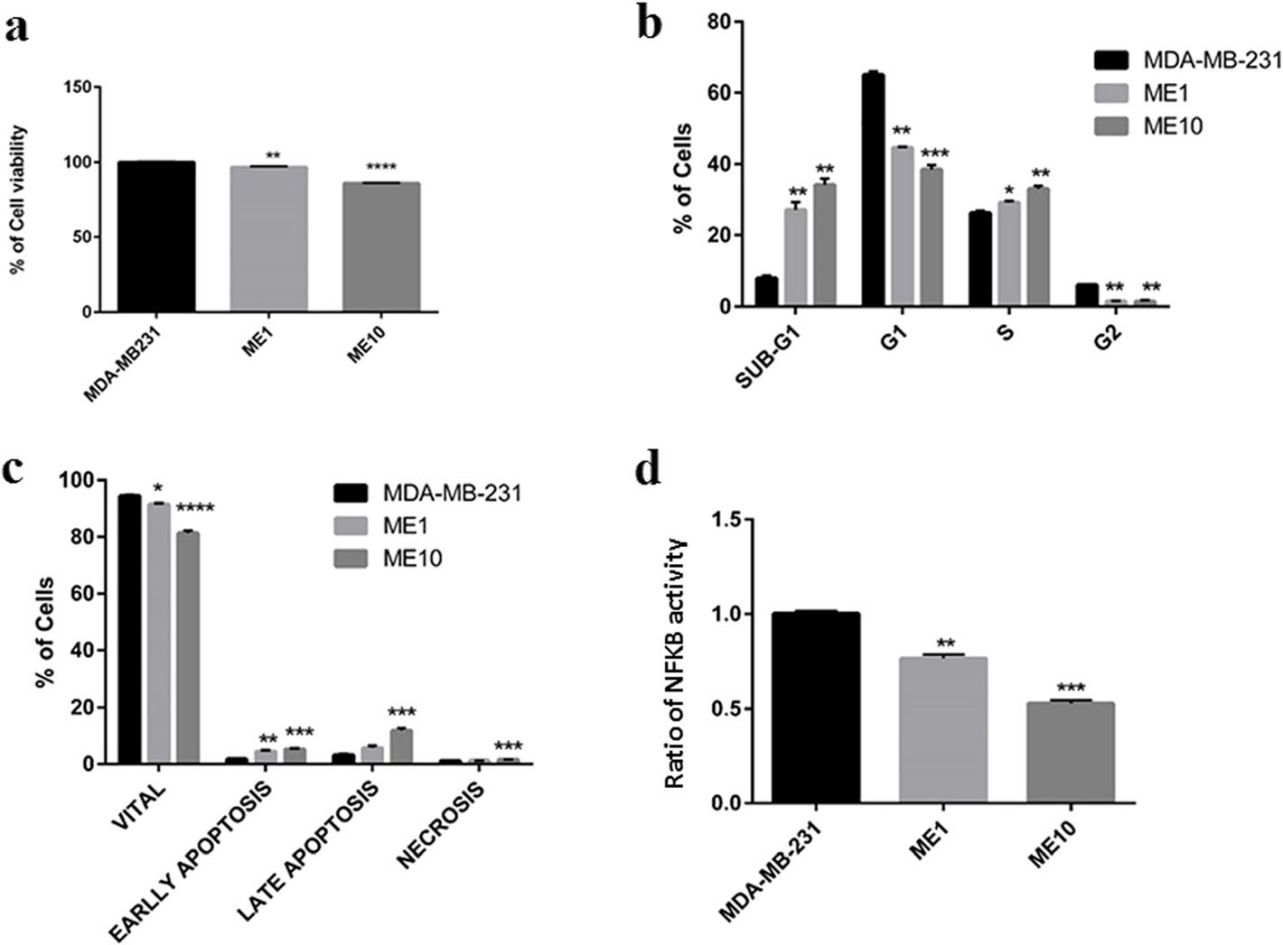
Assessing the anti-cancer effects of Etanercept as a TNF-α blocker. **a** MTT viability test in MDA-MB-231 cells following treatment of cells with 1 or 10 μg/ml Etanercept. Data represent mean ± sem for six replicates. ME1 and ME10 = MDA-MB-231 + 1 or 10 μg/ml Etanercept. **b** Analysis of cell cycle in MDA-MB-231 cells treated with 1 or 10 μg/ml Etanercept. Data represent mean ± sem of number of cells in each phase for 3 replicates. **c** Measurement of apoptosis using annexin-PI. Data represent mean ± sem of number of cells in each phase for 3 replicates. **d** Measurement of NFKB activity following treatment with Etanercept. Data are normalized to the activity of NFKB in MDA-MB-231 cells as control. ^*^P < 0.05 ^**^P < 0.01, ^***^P < 0.0001 . ME1 and ME10 = MDA-MB-231 + 1 or 10 μg/ml Etanercept

To further validate the in silico findings, in vitro validation experiments was followed in order to explore the effect of Etanercept, as a TNF-α inhibitor, on suppressing the invasive behavior of breast cancer cells. The viability assay revealed a significant decrease in cell survival following treatment of MDA-MB-231 cells with Etanercept in a concentration-dependent manner (1 and 10 μg/ml) (Fig. 1a). Cell cycle analysis revealed a significant reduction in the number of cells in G1 and G2 phases, and an increase in the number of cells in subG1 phase at both concentrations of Etanercept, collectively indicating an induction of apoptosis in cancer cells (Fig. 1b). Consistently, the Annexin-PI results displayed an elevated number of both early and late apoptotic cells upon treatment with 1 and 10 μg/ml of Etanercept, further confirming the induction of apoptosis (Fig. 1c). These results suggest thatof TNF-α inhibition is capable of reducing cancer cell survival rate.

The next step consisted of measuring p65 translocation to the nucleus of MDA-MB-231 cells, which revealed a significant reduction in the levels of active NFKB in the nucleus (*p*-value< 0.05) following treatment with Etanercept at 1 and 10 μg/ml, suggesting that Etanercept can also diminish the activity of NFKB in MDA-MB-231 cells (Fig. 1d).

### Cytotoxic effect of Etanercept is reduced in the presence of macrophages

Contrary to our findings on the inhibitory effects of Etanercept on the growth of breast cancer, there is also evidence demonstrating the inefficiency of Etanercept in suppressing the invasive behavior of tumors [24]. To address this discrepancy, following a closer inspection of MDA-MB-231 secretome within GSE51938 dataset revealed that GM-SCF is highly secreted by these cells [22]. This chemokine is an important activator of macrophages in inflamed tissues, also addressed by Su et al. [22]. These observations would likely justify the resistance to anti -TNF therapy.

**Fig. 2:**
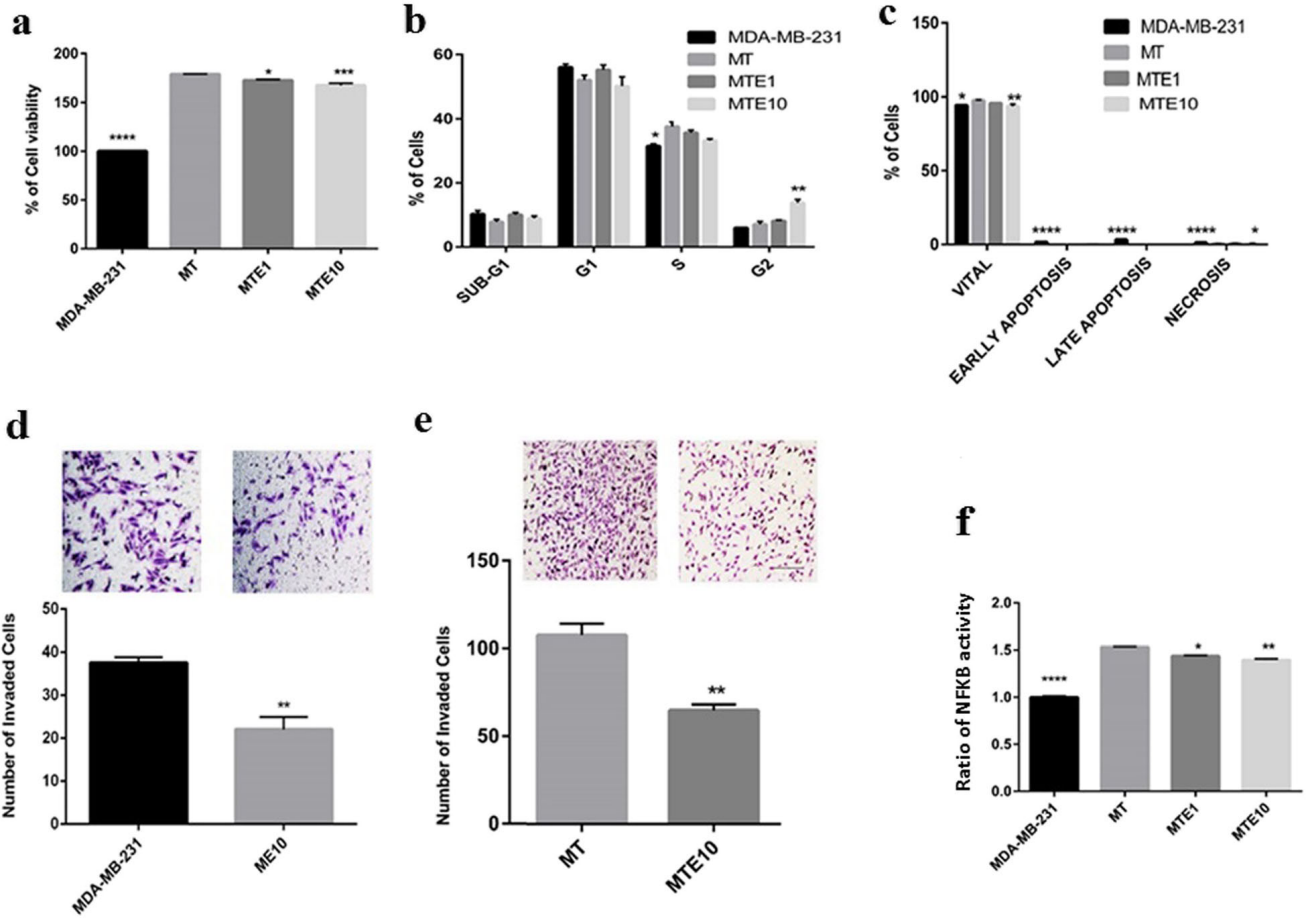
Assessing the anti-cancer effects of Etanercept in presence of macrophages (THP-1). **a** MTT viability test in MDA-MB-231 plus THP-1 cells following treatment of cells with 1 or 10 μg/ml Etanercept. Data represent mean ± sem for six replicates. MTE1 and MTE10 indicate THP-1 plus MDA-MB-231 plus Etanercept at concentrations 1 and 10 μg/ml, respectively. **b** Analysis of cell cycle in co-cultured MDA-MB-231 cells treated with 1 or 10 μg/ml Etanercept. Data represent mean ± sem of number of cells in each phase for 3 replicates. E1 = Etanercept at concentration 1 μg/ml, E10 = Etanercept at concentration 10 μg/ml. **c** Measurement of apoptosis using annexin-PI. Data represent mean ± sem of number of co-cultured cells in each phase for 3 replicates. **d**-**e** Matrigel invasion test to evaluate the effect of 10 μg/ml Etanercept (E10) on the invasive properties of MDA-MB-231 cells (ME10). MDA-MB-231 cells plus co-cultured THP-1 (MT) that were treated with 10 μg/ml of Etanercept (MTE10) are also shown. As illustrated, Etanercept decreased invasion of MDA-MB-231 cells. The anti-invasion efficacy diminished in presence of macrophages (T). ^*^ *P* < 0.05 ^**^P < 0.01, ^***^P < 0.0001. **f** Measurement of NFKB activity following treatment with Etanercept. MT = MDA-MB-231 plus THP-1 cells, MTE1 and MTE10 = THP-1 cells plus MDA-MB-231 plus Etanercept at concentrations 1 and 10 μg/ml, respectively

A co-culture system was used to measure several biological parameters. It was shown that co-culturing of MDA-MB-231 cells with THP-1, which were differentiated into M0 macrophages by low concentration of PMA, could significantly increase the viability of MDA-MB-231 cells and decrease the efficacy of Etanercept at 1 and 10 μg/ml in inhibiting survival, proliferation and apoptosis induction (Fig. 2a-c). However, 10 μg/ml of Etanercept was more potent than 1 μg/ml to cause G2/M arrest.

Invasion of cancer cells was increased in the presence of macrophages, and Etancercept was able to diminish this effect at 10 μg/ml (Fig. 2 d-e). Besides, the activity of NFKB in breast cancer cells was significantly augmented, and Etanercept was incapable of lowering this effect to the levels detected in the monocultures of cancer cells (Fig. 2f). These findings suggest that the interaction between cancer cells and macrophages maintains NFKB activity at a high level and inhibition of TNF-α alone would be insufficient to lower its activity.

**Fig. 3:**
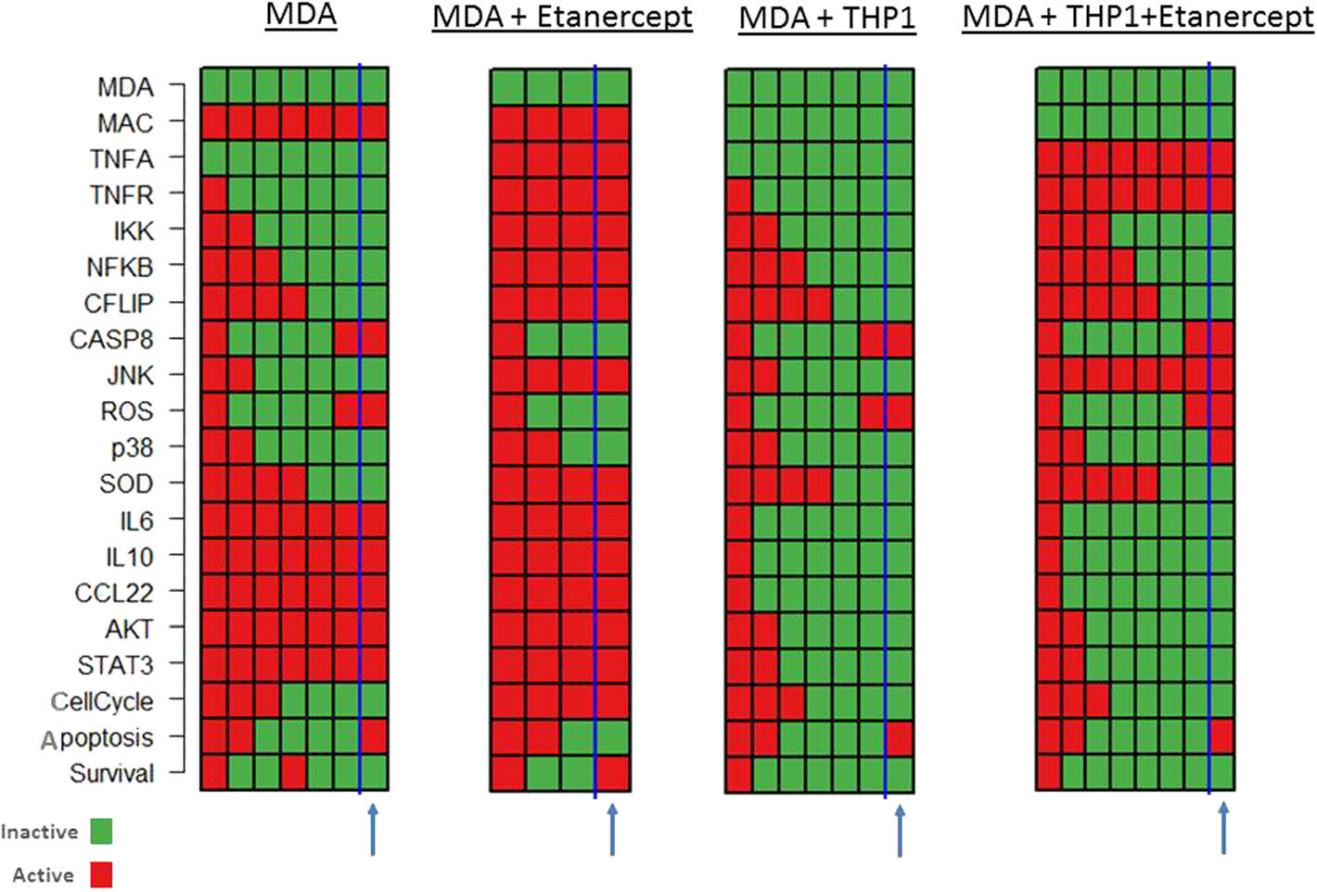
Literature-based illustration of TNF-α signaling pathway in the presence and absence of macrophages

The reduced efficacy of Etanercept in the presence of macrophages can also be predicted using the signaling network modeling. Hence, a network of TNF-α signaling pathway was constructed in the presence and absence of macrophage secretome using KEGG database and the those of Lu et als [19]. In constructing this signaling network, the crosstalk among core cellular pathways including NFKB, JAK-STAT and PI3K-AKT was taken into account based on the literature (Fig. 3). The interactions were then translated into a Boolean model to simulate TNF-α signaling in the presence and absence of macrophages. Asynchronous simulation model was used to predict cell fate; i.e. survival, proliferation and apoptosis when TNF-α pathway was active in cancer cells alone versus the condition where macrophages were considered (Supplementary file 1).

**Fig. 4:**
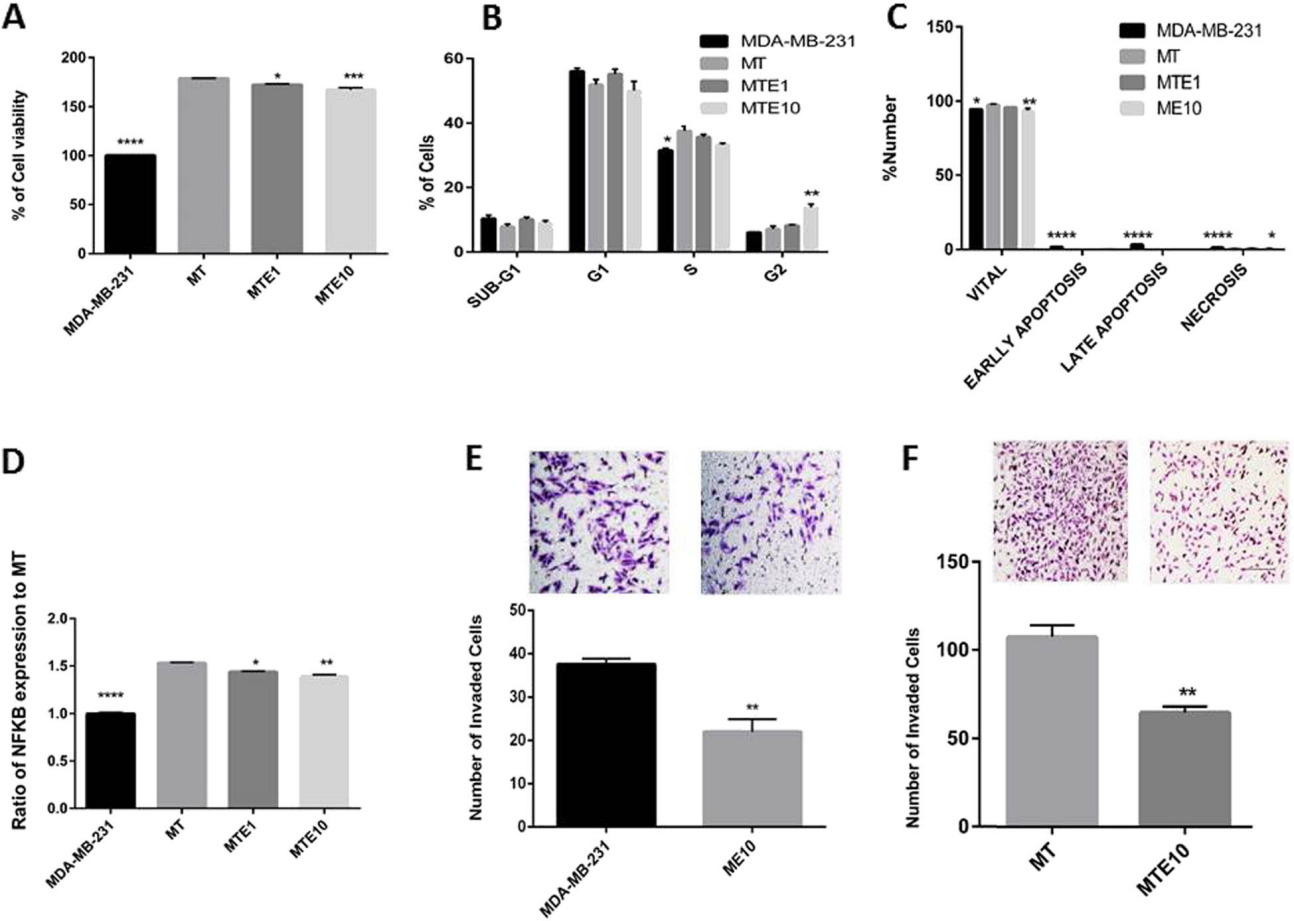
Dynamic modeling of pathway signaling components in the absence and presence of macrophages treated with Etanercept. Active and inactive genes are marked with green and red, respectively. The attractor i.e. the steady state is marked with a blue arrow

Attractor analysis (i.e. the steady state of the system) demonstrated that when breast cancer cells were modeled alone and treated with Etanercept (i.e. Macrophage and TNF-α state were set to 0), proliferation and cell survival were found in the OFF state while apoptosis was in the active state. However, in the presence of macrophages (Macrophage = 1) and Etanercept treatment (TNF-α = 0), apoptosis was in the inactive state (apoptosis = 0) and proliferation and survival were still active. These results suggest that Etanercept treatment alone is not effective when macrophage secretome activates additional signaling pathways in the tumors (Fig. 4).

### Effect of Etanercept on secretion of TNF-α, IL10 and CCL22 in macrophages

**Fig. 5:**
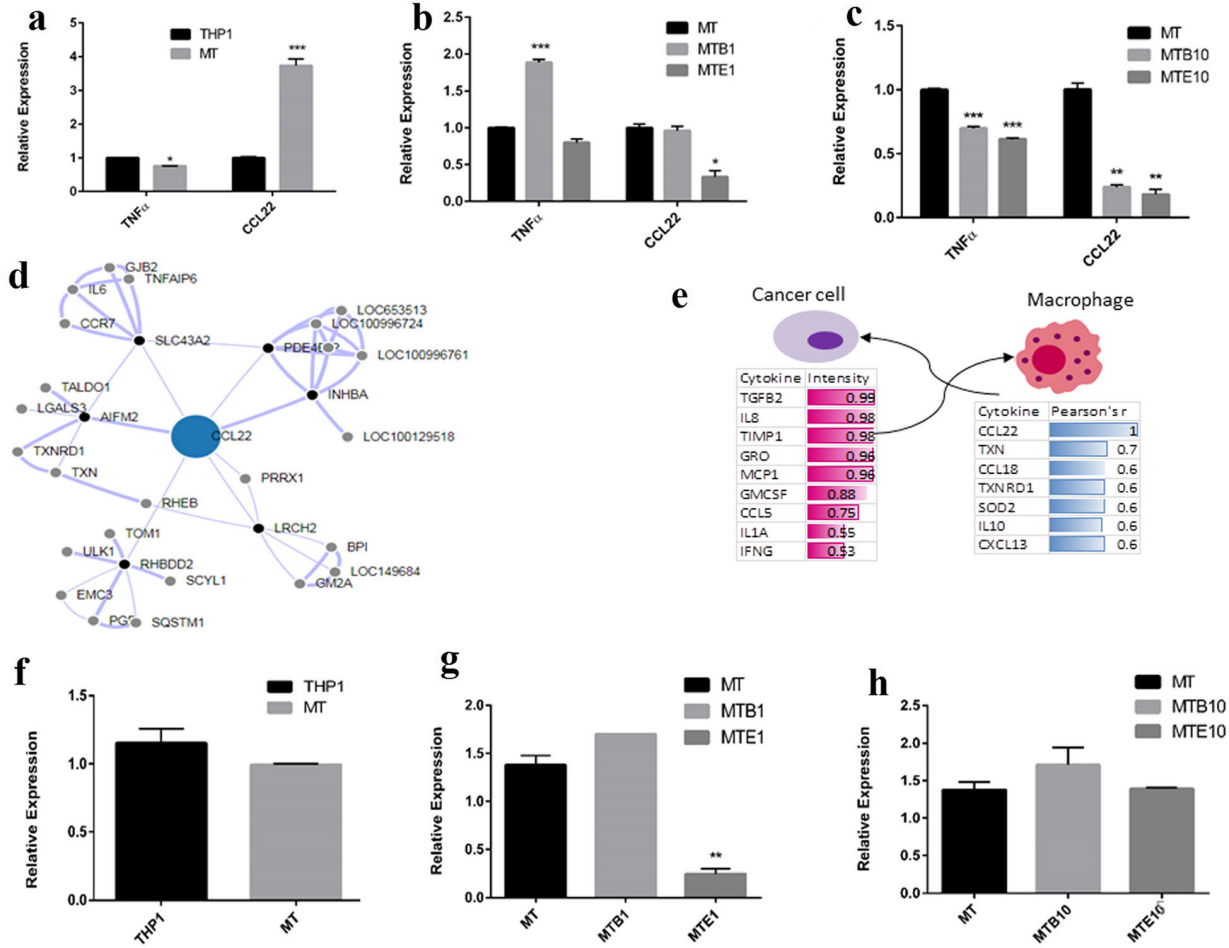
Analysis of expression of cytokines and chemokines in the CM-treated macrophages by RT-PCR. **a** Relative expression of CCL22 and TNF-α in THP-1 cells alone or treated with CM in presence or absence of Etanercept (E1 and E10 = 1 and 10 μg/ml Etanercept). MT = MDA-MB-231 + THP-1 cells. **b**, **c** Expression of CCL22 and TNF-α mRNA when THP-1 cells were treated with varying concentrations of Etanercept either alone or in presence of MDA-MB-231 condition media (CM). Due to statistically significant effect of the carrier vehicle of Etanercept on the expression of cytokines and chemokines, each concentration of drug is compared to the equivalent amount of vehicle as control. MT = MDA-MB-231 + THP-1 cells, MTB1 and MTB10 = MDA-MB-231 + THP-1 + Vehicle buffer at a concentrations equal to 1 and 10 μg/ml, MTE1 and MTE10 = MDA-MB-231 + THP-1+ Etanercept at concentartions 1 and 10 μg/ml. Data represent mean ± sem of three independent experiments. ΔCT are normalized to GAPDH. ^*^P < 0.05 ^**^P < 0.01, ^***^P < 0.0001. **d** Co-expression network obtained from co-expression analysis of CCL22 in immune navigator online tool. THP-1 was chose as cell line and correlation coefficient > 0.6 was considered significant. **e** Interaction between cancer cells and macrophages through secretion of co-expressed cytokines. The highly secreted cytokines in MDA-MB-231 were derived from GSE51938. For macrophages, highly correlated genes with correlation coefficient > 0.6 are shown. **f**, **g**, **h** Expression of IL10 in THP-1 cells alone or treated with CM in presence or absence of Etanercept (E1 and E10 = 1 and 10 μg/ml Etanercept). MT = MDA-MB-231 + THP-1 cells, MTB1 and MTB10 = MDA-MB-231 + THP-1 + Vehicle buffer at a concentrations equal to 1 and 10 μg/ml, MTE1 and MTE10 = MDA-MB-231 + THP-1+ Etanercept at concentartions 1 and 10 μg/ml. Data represent mean ± sem of three independent experiments. ΔCT are normalized to GAPDH. **P < 0.01

In order to explore the changes in the function of macrophages following treatment with CM or Etanercept, the effect of Etanercept on the expression levels of TNF-α as well as CCL22, a chemokine marker of M2 phenotype of tumor-associated macrophages contributing to aggressive features of tumors, were measured [25]. It was revealed that the expression level of CCL22 in macrophages grown in CM for 48 h was increased compared to cells treated with fresh medium alone, while the same condition slightly decreased the expression of TNF-α (Fig. 5a). In this regard, Etanercept at 1 and 10 μg/ml significantly diminished the expression level of both factors (Fig. 5 b, c).

To extend the list of other co-expressed proteins with CCL22 in CM-treated macrophages, gene co-expression network analysis in THP-1 cells was utilized (Fig. 5d) using Immuno-Navigator web tool (Pearson’s correlation coefficient < 0.6) [26]. Thirty nine genes were found to be co-expressed with CCL22 (Supplementary file 2). Among the top co-expressed genes were several cytokines including IL10 along with chemokines such as CCL18 and CXCL13 as well as anti-oxidant enzymes such as SOD2, TXN and TXNRD1 (Fig. 5e). From these top correlated genes, we determined the expression of IL10 following co-culturing and treatment with Etanercept by qPCR. Co-culturing of differentiated macrophages with MDA-MB-231 cells did not significantly alter the expression level of IL10, likely due to the short co-culturing period used in the study (Fig.5f). However, Etanercept decreased the expression of IL10 at both concentrations of 1 and 10 μg/ml compared to the control vehicle (Fig. 5g,h). These results demonstrated that Etanercept can affect the secretory profile in addition to the function of tumor-associated macrophages; however to a lesser extent.

## Discussion

NFKB is highly active in the invasive breast cancer cells resulting in the secretion of a number of cytokines, a condition similar to that of cells treated with TNF-α. These data fueled our initial hypothesis that using clinically available TNF-α inhibitors would be a smart approach to alter the secretion profile of invasive breast cancer cells, and thus motivated us to assess the inhibitory effect of Etanercept, a TNF-α inhibitor, to suppress proliferation and survival of breast cancer cells. Our hypothesis is also supported by the findings of other studies indicating the efficacy of Etanercept, a soluble receptor, to capture and neutralize circulating TNF-α in breast cancer treatment [18, 27]. The present study showed that inhibition of TNF-α signaling by Etanercept results in a reduction in the activity of NFKB transcription factor, leading to reduced survival rates of MDA-MB-231 cells, and induced cell cycle arrest and apoptosis.

Notably, the effect of anti-TNF therapies is still controversial with conflicting outcomes at patient level. In this regard, it has been shown that tumor microenvironment exerts profound effects not only on the behavior of cancer cells but also on the efficacy of treatments that has to be taken into account when evaluating the effect of novel drugs [28]. To address this issue, using publicly available proteomic data of MDA-MB-231 secretome, a high level of Granulocyte-Monocyte Stimulating Factor (GM-CSF) expression was detected in the secretome, which modulate activation of tumor associated macrophages. Accordingly, our findings on cell cycle analysis, annexin/PI and matrigel assays further confirmed that when co-cultured with MDA-MB-231 cells, macrophages reduce the anti-proliferative and anti-invasive effects of Etanercept in cancer cells. These findings suggested that the anti-cancer effect of Etanercept can be diminished or abrogated by resident macrophages in the tumor site, which would in turn suggest a role for the microenvironment in treatment efficiency.

We also observed that in the co-culture condition, Etanercept does not reduce the activity of NFKB to the extent observed in breast cancer cells alone. These results underscore the importance of stromal cell secretome in resistance to the targeted therapies. Tumor associated macrophages are important components of tumor stroma, which link inflammation and cancer progression by secreting various cytokines. The secreted cytokines from macrophages can trigger PI3K/AKT/mTOR signaling pathway [29] and also lead to the activation of NFKB as well as STAT3 in cancer cells [30–32]. Interaction between NFKB and STAT3 is a key mediator of crosstalk in the tumor inflammatory microenvironment [33]. STAT3 is constitutively activated in triple negative breast cancer, and ample evidence imply that persistently- activated STAT3 maintains constitutive NFKB activity [34, 35]. Collectively, these results suggest that the presence of macrophages in tumor tissues potentiates the activity of NFKB resulting in subsequent resistance to anti-TNF therapies. In other words, the activated signaling pathways can decrease the reliance of NFKB activation solely on TNF-α. This mechanism may apparently reduce the efficacy of TNF-α inhibition strategy.

Moreover, we used Boolean network modeling to simulate the effect of Etanercept in the presence and absence of macrophages. This modeling provides useful insights to confirm our findings that in the presence of macrophage-associated cytokines and chemokines, activation of JAK-STAT and PI3K-AKT pathways can restore survival and proliferation of tumor cells to compensate the decreased activity of NFKB by Etanercept.

We were also interested to assess the effect of Etanercept on the secretion profile of macrophages that were grown in the CM. We showed that Etanercept reduces the transcript levels of TNF-α, IL10 and CCL22, all of which are associated with tumor progression. Thus our results imply that in addition to inhibiting the aggressive behavior of tumor cells within the context of microenvironment, Etanercept is able to alter the activity of tumor associated macrophages.

As a whole, our data suggest that evaluating the anticancer effect of drugs without considering the effect of stromal cells may lead to failure of drug candidates in the later clinical stages. An integrated use of high through-put data available in the literature and systems biology would allow predicting the effect of drugs in the presence of stromal cells. These data should further be validated by in vivo experiments.

## Conclusions

In summary, we concluded that the anticancer efficacy of Etanercept as a TNF-Α blocker is influenced by the secretome of the co-cultured macrophages. These results suggest an important effect of stromal cells on the efficacy of targeted drugs in cancer treatment.

## Supplementary information

**Additional file 1.**

**Additional file 2.**

## Data Availability

The source code for Boolean network construction and highly correlated genes are provided as supplementary information.

## Abbreviations

GM-CSF: Granulocyte-monocyte colony stimulating factor
LINCS: Library of Integrated Network of Cellular Signaling
TNF1-α: Tumor Necrosis Factor alpha
FADD: Fas-Associated protein with Death Domain
JNK: C-Jun N-terminal Kinase
EMT: Epithelial-Mesenchymal Transition
