# Correction to: The efficacy of etanercept as anti-breast cancer treatment is attenuated by residing macrophages

**DOI:** 10.1186/s12885-020-07641-3

**Published:** 2020-11-20

**Authors:** Elnaz Shirmohammadi, Seyed-Esmaeil Sadat Ebrahimi, Amir Farshchi, Mona Salimi

**Affiliations:** 1 School of Pharmacy, International Campus, Tehran University of Medical Sciences, Tehran, Iran; 2 Biopharmaceutical Research Center, AryoGen Pharmed Inc., Alborz University of Medical Sciences, Karaj, Iran; 3 Physiology and Pharmacology Department, Pasteur Institute of Iran, P.O. Box: 13164, Tehran, Iran

**Correction to: BMC Cancer 20, 836 (2020)**

**https://doi.org/10.1186/s12885-020-07228-y**

Following publication of the original article [1], the authors report the following errors in their article:

1. In the Background section, the last sentence of the first paragraph has been corrected to the following: The secreted factors reprogram the surrounding stroma with the aim of neutralizing the impact of various *inducers* disrupting the survival of the cancer cells.
  It was originally published as: The secreted factors reprogram the surrounding stroma with the aim of neutralizing the impact of various *intruders* disrupting the survival of the cancer cells.

2. Figure 1 legend is incorrect. ***P < 0.001 (not 0.0001)

3. Figure 2 legend is incorrect. ***P < 0.001 (not 0.0001)

4. Updated Fig. 3 and Fig. 4 are published in this correction article.

**Fig. 3:**
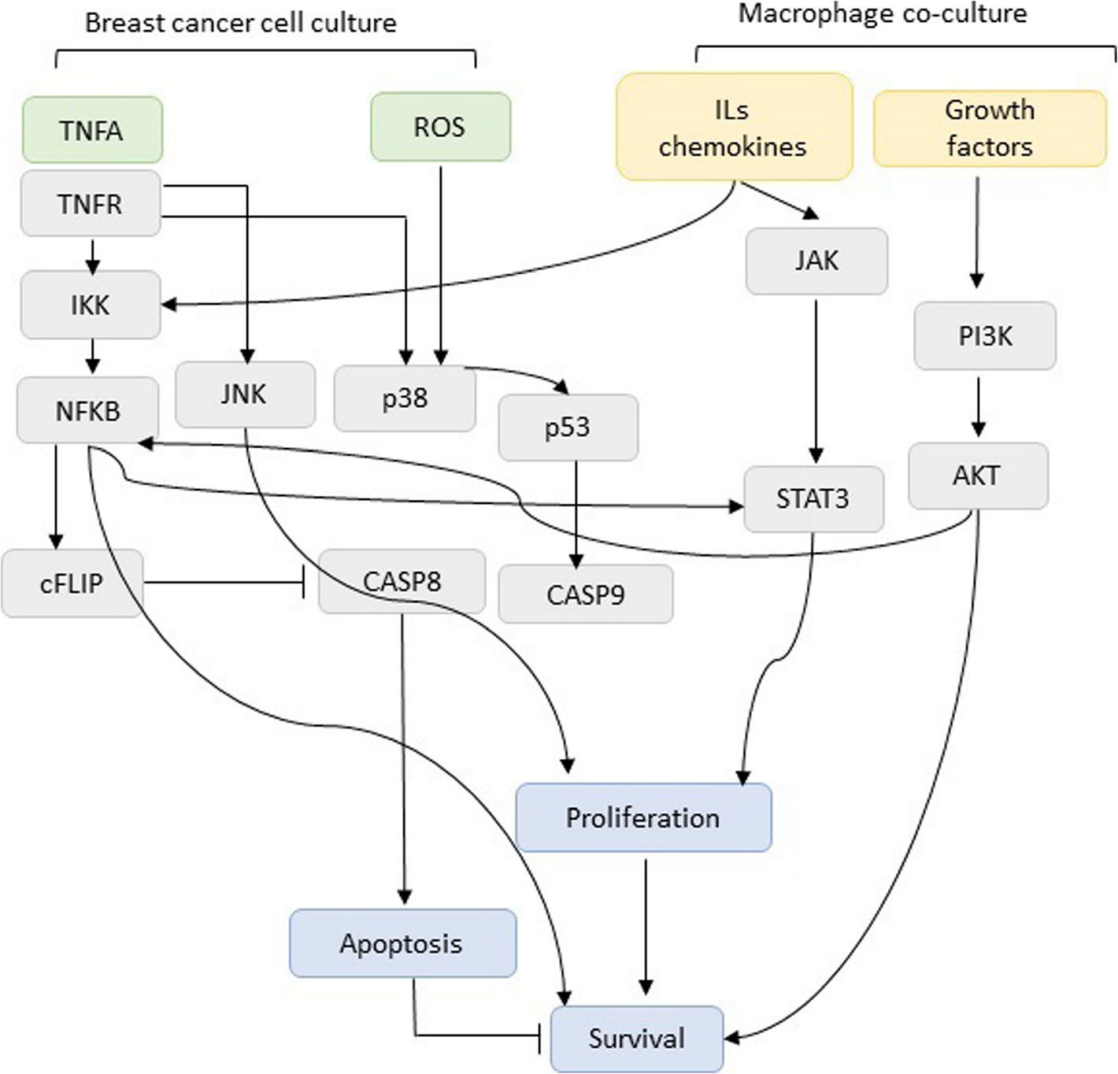
Literature-based illustration of TNF-α signaling pathway in the presence and absence of macrophages

**Fig. 4:**
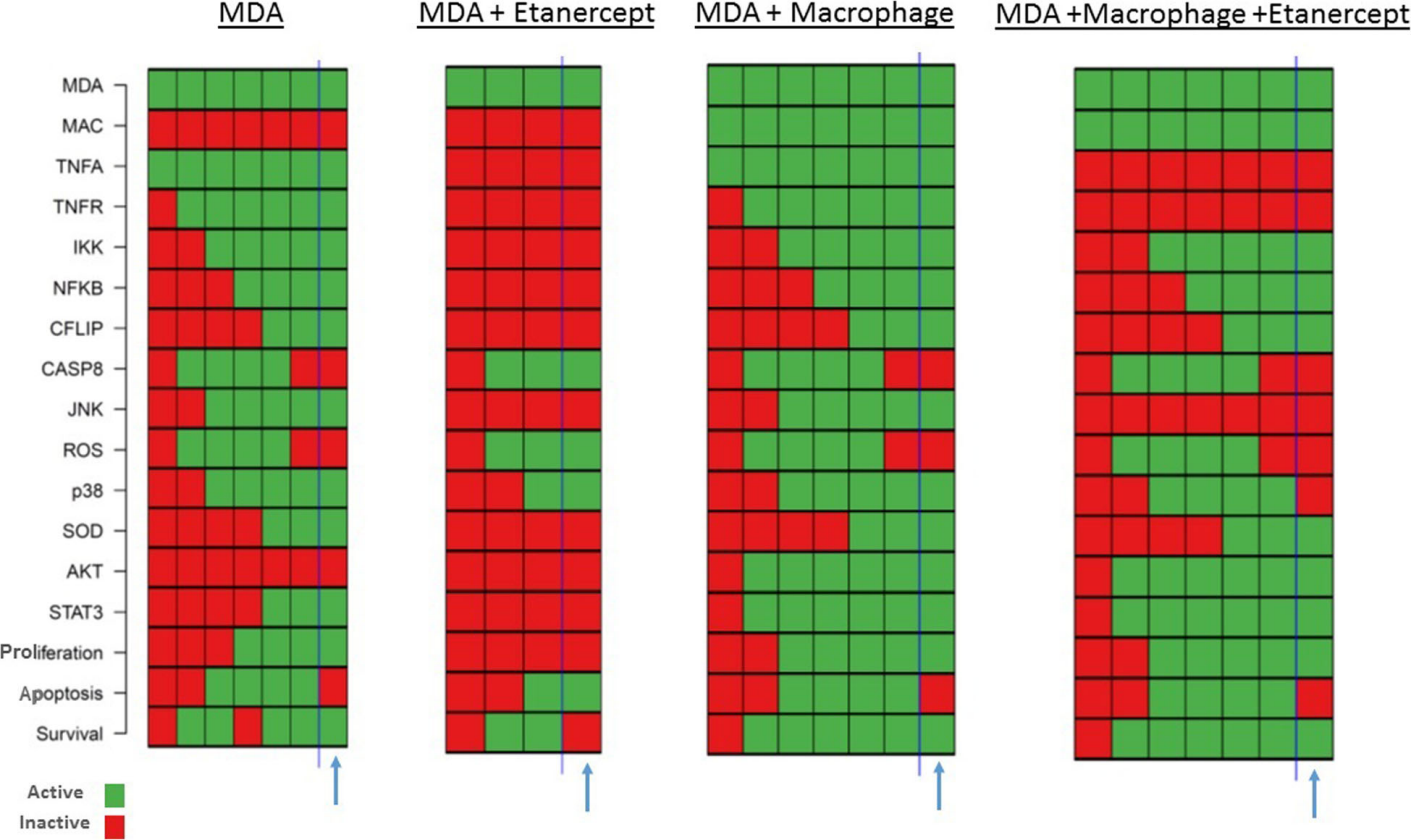
Dynamic modeling of pathway signaling components in the absence and presence of macrophages treated with Etanercept. Active and inactive genes are marked with green and red, respectively. The attractor i.e. the steady state is marked with a blue arrow

5. Figure 5 legend is incorrect. ***P < 0.001 (not 0.0001)
